# Open Hill-Sachs remplissage associated with glenoid bone block: a single deltopectoral approach

**DOI:** 10.1016/j.xrrt.2026.100706

**Published:** 2026-03-02

**Authors:** Thomas Boissinot, Yves Lefebvre

**Affiliations:** a Centre Hospitalo-Universitaire La Milétrie, Poitiers, France; b Institut de l’épaule, Strasbourg, France

**Keywords:** Hill-Sachs, Remplissage, Open, Latarjet, Eden-Hybinette, Glenoid track, Shoulder instability, Bipolar bone loss

Glenohumeral stabilization using a Latarjet procedure has proven to be highly effective, yet a recurrence rate of up to 18% has been reported in the literature.^22^ Several factors appear to increase the risk of recurrent instability after a Latarjet procedure: hyperlaxity, bipolar bone loss, off-track Hill-Sachs lesion, epilepsy, bilateral glenohumeral instability, and revision surgery cases.^15^^,^^19^^,^^29^^,^^36^^,^^39^

In the event of failure, so-called “salvage procedures” remain a technical challenge, and clinical outcomes are poorly described in the literature. According to the systematic review by Baur et al, a glenoid bone graft following the Eden-Hybinette technique is most frequently performed.^4^ However, there is variation in graft origin (iliac crest autograft, distal tibial allograft), fixation method (screws, suture buttons), and surgical approach (arthroscopic vs. open). In such cases, the risk of recurrence can reach 44%.

To enhance stability in high-risk patients, some authors recommend adding a remplissage of the Hill-Sachs lesion to the Latarjet procedure as a first-line treatment.^13^

Since Ranne's 2013 description, few studies have reported on this dual procedure^34^ (Table I). Some authors perform open Latarjet with arthroscopic remplissage,^2^^,^^3^^,^^27^ requiring 2 surgical setups and possibly changing patient positioning, potentially increasing operative time and cost.

**Table I:** Summary of published studies reporting combined glenoid bone block procedures associated with Hill-Sachs remplissage in the treatment of anterior shoulder instability.

Date	Author	Type of article	Context	Indication	N	Technique	FU	Recurrence
2024	Boileau et al.	Prospective cohort	First-line	GBL: >10% HSL: Calandra 3	41	Arthroscopic Latarjet Arthroscopic Remplissage	24	7%
2024	Arora et al.	Retrospective cohort	First-line	GBL: >15% HSL: large, off-track	11	Open Eden Hybinette Arthroscopic Remplissage	12	0%
2022	Callegari et al.	Cadaver study	-	GBL: 20% HSL: off-track	9	Distal tibia allograft Remplissage	-	-
2022	Abboud et al.	Case series	First-line	GBL: >25% HSL: large, off-track	5	Open Latarjet Arthroscopic remplissage	31	%
2019	Boileau et al.	Retrospective cohort	Revision	HSL: large	2	Arthroscopic Eden-Hybinette Arthroscopic Remplissage		
2017	Saliken et al.	Technique article	First-line	BBL ISI score >3	-	Arthroscopic Latarjet Arthroscopic Remplissage	-	-
2016	Katthagen et al.	Technique article	First-line	GBL: >25% HSL: large, off-track	-	Open Latarjet Arthroscopic Remplissage	-	-
2013	Ranne et al.	Case report	First-line	GBL: large HSL: large, engaging	1	Open Latarjet Arthroscopic Remplissage	36	No

*BBL*, bipolar bone loss; *FU*, follow-up; *GBL*, glenoid bone loss; *HSL*, Hill-Sachs lesion; *ISI score*, Instability Severity Index Score; *N*, number of patients.

Therefore, to our knowledge, all authors having studied this dual procedure used arthroscopic remplissage, either with arthroscopic or open bone block. Initially described as an open technique by Connolly, remplissage involved transferring the infraspinatus tendon into the humeral defect through a posterior approach.^14^ It was later adapted to arthroscopy by Wolf^32^ and further modified by Koo.^28^ As a result, open remplissage is now rarely performed.^1^^,^^14^

In our experience, this technique is performed for patients at high risk of recurrence as follows. In the primary setting, eligibility requires at least 2 of the following risk factors: a large Hill–Sachs lesion, critical glenoid bone loss, a medical history of epilepsy, or hyperlaxity. In the revision setting for recurrent instability, one of these factors is considered sufficient to warrant the addition of a remplissage procedure.

This technical article aims to describe feasibility of an open remplissage during an open Latarjet or free bone-block procedure through a single deltopectoral approach.

## Technique

### Case presentation

**Figure 1:**
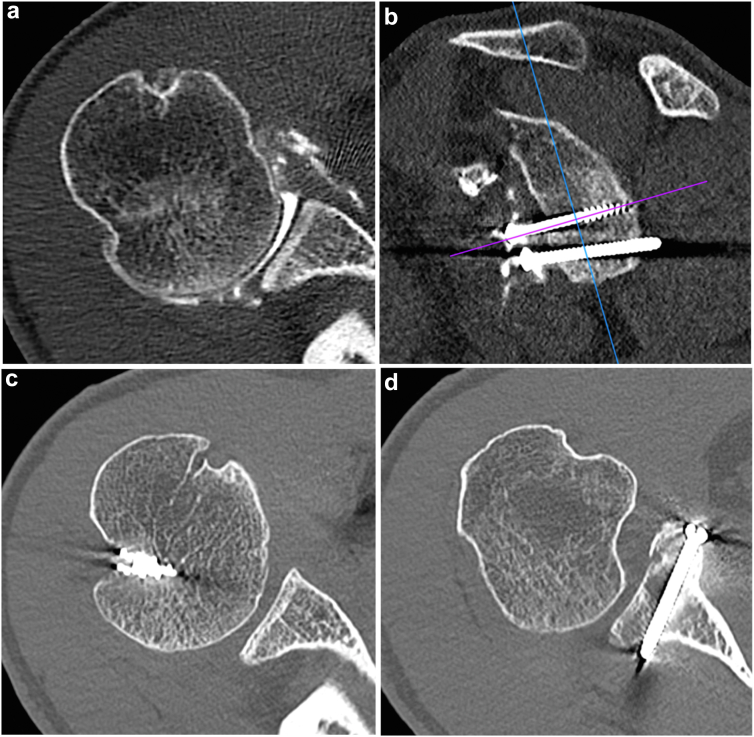
Pre-operative CT scan (**a** and **b**) showing bone block osteolysis and moderate Hill-Sachs lesion (**a** and **b**). Post-operative CT scan at 4 months follow-up showing bone block consolidation (**c** and **d**). *CT*, computed tomography.

**Figure 2:**
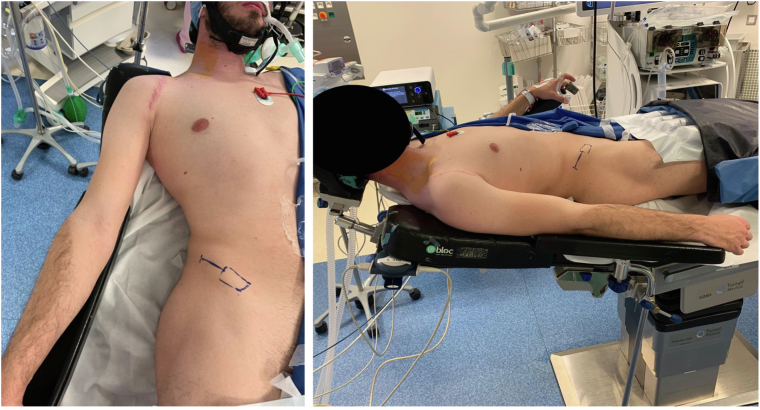
Patient positioned in the lazy beach chair setup with the operative arm placed on an armrest in slight abduction. In this case, an iliac crest harvest is planned for an Eden-Hybinette bone block procedure.

**Figure 3:**
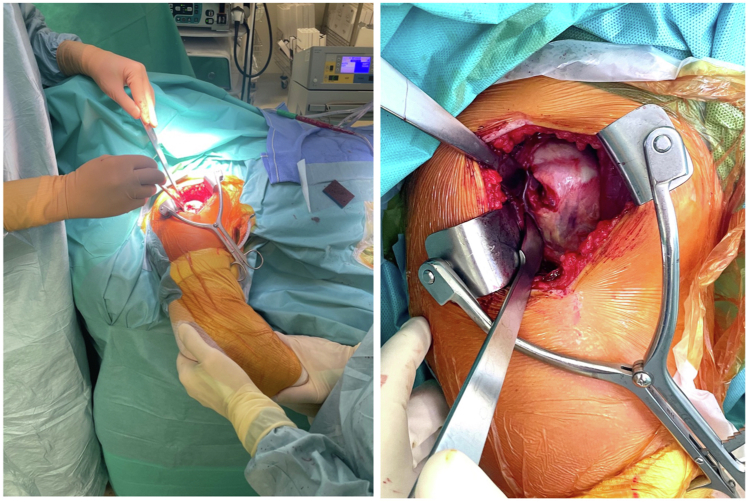
The upper limb is positioned in internal rotation and extension to expose the posterior aspect of the rotator cuff, allowing a longitudinal incision over the Hill-Sachs lesion.

**Figure 4:**
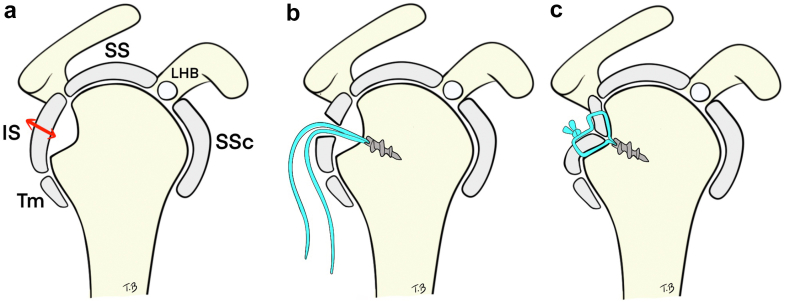
(**a**) Exposure of the Hill-Sachs lesion through a longitudinal incision of the posterior rotator cuff. (**b**) Anchor placement after débridement of the lesion. (**c**) Suture passage through the cuff and fixation using mattress stitches to achieve remplissage of the defect. *IS*, infraspinatus; *LHB*, long head of the biceps; *SS*, supraspinatus; *SSc*, subscapularis; *Tm*, teres minor.

**Figure 5:**
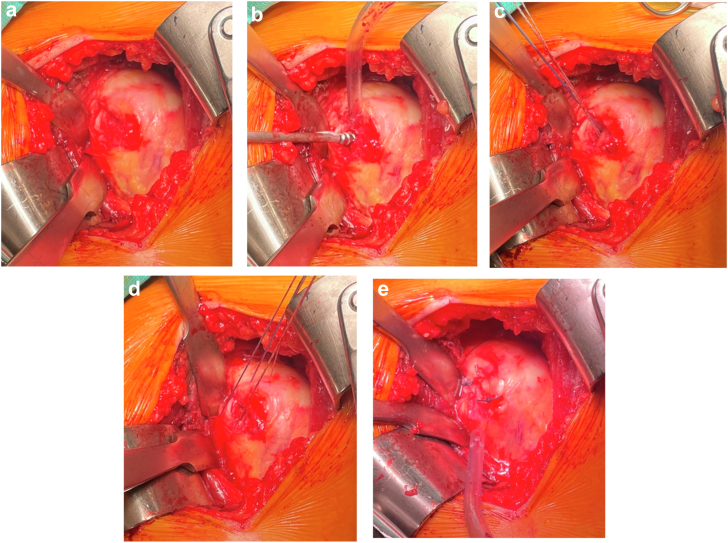
(**a**) Incision of the posterior cuff allowing visualization of the Hill-Sachs lesion. (**b** and **c**) Anchor placement after bone debridment. (**d**) Passage of sutures through the posterior cuff on both sides of the incision. (**e**) Creation of 2 mattress sutures to fill the defect and close the incision.

**Figure 6:**
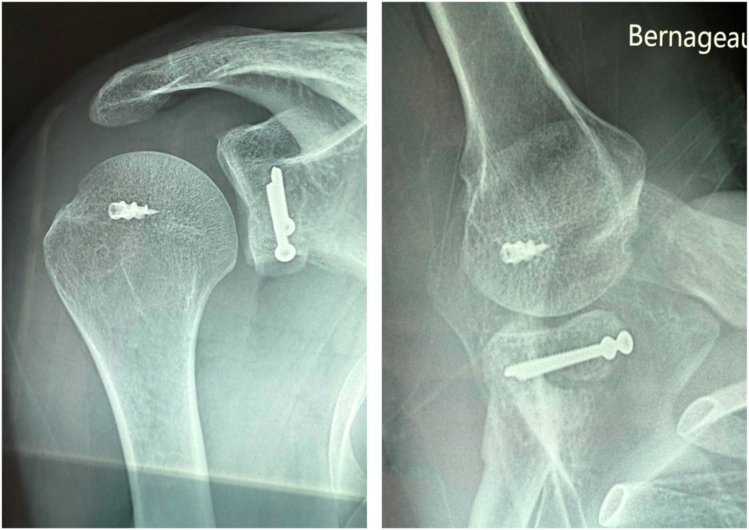
Post-operative radiographs of an anterior glenoid bone block combined with Hill-Sachs remplissage.

A 22-year-old male, right dominant, manual laborer presented with right shoulder pain and recurrent anterior instability one year after an open Latarjet procedure (Figure 1, Figure 2, Figure 3, Figure 4, Figure 5, Figure 6). Apprehension test and relocation test were positive. There was no sign of hyperlaxity. Computed tomography (CT) scan showed bone block osteolysis, critical glenoid bone loss, and a Grade II Hill-Sachs lesion according to Calandra's classification^10^ (Fig. 1).

In the setting of revision surgery and significant bipolar bone loss, a combined procedure, consisting of an iliac crest bone block associated with an open Hill-Sachs remplissage through a single deltopectoral approach.

### Surgical procedure

The procedure is demonstrated in Supplementary Video S1. It is performed under general anesthesia. The patient is placed in a “lazy beach chair” position with approximately 20° of tilt (Fig. 1). The operative upper limb should remain free in the sterile field. It is critical to ensure that the arm can be freely mobilized, especially allowing for full internal rotation of the humerus to expose its posterior aspect during surgery.

In primary Latarjet, remplissage is performed after coracoid osteotomy and before fixation of the bone block. This increases internal rotation and anterior translation of the humerus, thus improving posterior cuff exposure.

In revision of a failed Latarjet with a free bone graft, the osteotomy has already been performed in the initial surgery. The remplissage is done prior to the free graft fixation.

From the deltopectoral interval, the deltoid is mobilized digitally around the rotator cuff to the posterior humeral aspect. A curved retractor such as a Hohmann is placed under the deltoid to retract it entirely. With internal rotation and extension, the posterior cuff is exposed.

After identifying the Hill-Sachs defect on pre-operative imaging, it is located intraoperatively by palpation under the tendon. A longitudinal infraspinatus split and capsulotomy are made to expose the defect (Fig. 2). A Gelpi retractor may be used to improve visualization.

The bone is carefully débrided to optimize healing. A double-loaded metal anchor (Corkscrew, Athrex) is placed in the defect. The 4 suture limbs are passed through the capsule and tendon on each side of the incision. Internal rotation is reduced to a neutral/slight internal rotation position (approximately 0–20°) to relax the posterior cuff and facilitate suture tying without overtensioning the tendon. This simultaneously achieves infraspinatus capsulotenodesis into the humeral defect and side-to-side closure of the tendon-capsule incision (Figs. 3 and 4). Once remplissage is complete, the arm is returned to external rotation, and the bone block is fixed (Fig. 5).

Post-operatively, the upper limb is immobilized in internal rotation for 30 days. Activities of daily living are allowed. Passive physical therapy begins immediately, with active therapy starting at one month. Noncontact sports may resume at 4 months and contact sports at 6 months.

### Clinical and radiological outcome

**Figure 7:**
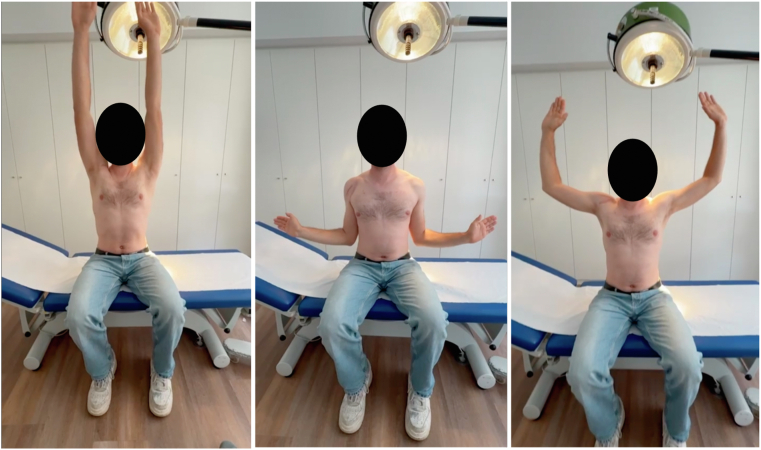
Post-operative range of motion at 4 months follow-up.

At the 4-month follow-up, the patient reported no pain and satisfying range of motion (Fig. 7). CT scan showed good positioning and bone block consolidation (Fig. 1).

## Discussion

This surgical technique allows for Hill-Sachs remplissage in conjunction with open bone-block stabilization through a deltopectoral approach.

Historically, humeral bone defects in shoulder instability have been managed using techniques such as remplissage.^12^ The glenoid track concept offers a more comprehensive approach to bipolar bone loss.^18^^,^^25^^,^^26^^,^^38^ Multiple studies have shown that off-track Hill-Sachs lesions are an independent risk factor for poor outcomes following Latarjet.^29^^,^^39^ When addressing major bipolar bone loss, the Latarjet procedure alone may not be sufficient to restore glenohumeral stability,^8^^,^^31^ especially in subjects with low-volume coracoid.^7^ A cadaveric study by Patel et al demonstrated that the Latarjet is indeed less effective in the presence of large Hill-Sachs defects, suggesting an additional humeral procedure may be required.^30^

Indeed, Calvo et al reported that Latarjet alone failed to convert off-track lesions to on-track in 12% of cases, with a significantly higher recurrence risk.^11^ Therefore, they recommend adding a remplissage to Latarjet procedures in cases of off-track Hill-Sachs lesions and glenoid bone loss above 25%.^16^^,^^17^

After the initial report of combined arthroscopic Latarjet, Bankart repair, and remplissage in 2017, Boileau et al published a 2-year follow-up study in 41 patients.^6^^,^^35^ Patients had glenoid bone loss ≥10% and large Hill-Sachs defects. The recurrence rate was only 7%. However, arthroscopic Latarjet presents challenges: a steep learning curve,^37^ high costs,^33^ and uncertain functional benefits.^23^

Recurrent instability after Latarjet is another high-risk scenario.^31^ Flurin et al reported a 14% recurrence at 3 years.^21^ Causes may include hardware failure, graft resorption, or underlying conditions (epilepsy, hyperlaxity, collagen disorders) or persistent on-track defects despite restored glenoid surface. Revision techniques typically include free bone grafting via iliac crest autograft (Eden-Hybinette) or allografts (iliac crest, tibia, clavicle).^4^^,^^9^ Outcomes are acceptable, but function remains suboptimal, and recurrence risk is higher than in primary procedures. In a study of 16 patients undergoing second-line iliac crest grafting for Latarjet failure, Ernstbrunner et al reported 7 recurrences.^20^ To improve stability and reduce recurrence in these difficult revision cases, remplissage may be combined with glenoid bone grafting.

In a series reporting arthroscopic Eden-Hybinette after Latarjet failure, Boileau et al included 3 patients who underwent concurrent Hill-Sachs remplissage for large defects with no recurrence of instability.^5^

Currently, the outcomes of open remplissage in shoulder anterior instability have not been reported, with no comparison to the arthroscopic technique. While both share similar biomechanical goals, open remplissage allows for straightforward dual procedure when performing open bone block.

On the other hand, open and arthroscopic Latarjet have been more extensively compared in the literature. Deng et al meta-analysis concluded that open procedure has better screw positioning, a lower revision rate, and a shorter learning curve, making it well suited to combine with an open remplissage.^16^

This technique has certain limitations. Exposing the posterior cuff by retracting the deltoid and soft tissues can be challenging in overweight patients. Deltopectoral access to the posterior humerus also requires adequate internal rotation, which can be hindered by stiffness. Finally, post-operative external rotation limitation has been described after both Latarjet and Hill-Sachs remplissage.^24^ Combining these 2 may further reduce motion, though this remains to be studied.

Clinical outcomes need to be studied to better define the indications for this technique, taking into account parameters such as, but not limited to, glenoid track, coracoid size, and relevant patient demographic factors.

## Conclusion

This open remplissage technique through a deltopectoral approach can be combined with either a coracoid or free glenoid bone-block procedure. It offers an additional stabilizing element in cases of major instability due to bipolar bone loss, patient characteristics (epilepsy, hyperlaxity), or revision context.

Performed through a single open approach, it represents a simple method to enhance shoulder stability in high-risk recurrence scenario.

## Disclaimers:

Funding: No funding was disclosed by the authors.

Conflicts of interest: The authors, their immediate families, and any research foundations with which they are affiliated have not received any financial payments or other benefits from any commercial entity related to the subject of this article.

## Supplementary Data

Video 1 Step by step open remplissage technique using a deltopectoral approach.

## References

[1] Abdelhady A.M. (2010). Neglected anterior shoulder dislocation: open remplissage of the Hill-Sachs lesion with the infraspinatus tendon. Acta Orthop Belg.

[2] Abboud J., Moussa M.K., Boushnak M.O., Rahal M.J.H., Robial N. (2022). Belt and suspender technique for bipolar bone loss in shoulder instability. JSES Rev Rep Tech.

[3] Arora M., Shukla T., Vala P. (2024). Managing severe bipolar bone loss in athletes: a comprehensive approach with open Latarjet and arthroscopic remplissage. J Orthop.

[4] Baur A., Satalich J., O'Connell R., Vap A. (2024). Surgical management of recurrent instability following Latarjet procedure - a systematic review of salvage procedures. Shoulder Elbow.

[5] Boileau P., Duysens C., Saliken D., Lemmex D.B., Bonnevialle N. (2019). All-arthroscopic, guided Eden-Hybbinette procedure using suture-button fixation for revision of failed Latarjet. J Shoulder Elbow Surg.

[6] Boileau P., Ranieri R., Lavoué V., Saliken D. (2024). Results of combined all-arthroscopic Latarjet with Hill-Sachs remplissage for significant bipolar glenohumeral bone loss. J Shoulder Elbow Surg.

[7] Boden S.A., Godshaw B.M., Hughes J.D., Musahl V., Lin A., Lesniak B.P. (2024). Preoperative imaging predicts coracoid graft size and restoration of the glenoid track in Latarjet procedures. JSES Int.

[8] Brandariz R.N., Gorodischer T.D., Pasqualini I., Rossi L.A., Tanoira I., Ranalletta M. (2021). The latarjet procedure without remplissage is effective to restore stability in athletes with glenoid bone defects greater than 25% and off-track hill-sachs lesions. Arthroscopy.

[9] Buda M., D'Ambrosi R., Bellato E., Zanini A., Facchini R.M., Zatti G. (2021). Failed Latarjet procedure: a systematic review of surgery revision options. J Orthop Traumatol.

[10] Calandra J.J., Baker C.L., Uribe J. (1989). The incidence of Hill-Sachs lesions in initial anterior shoulder dislocations. Arthroscopy.

[11] Calvo C., Calvo J., Rojas D., Valencia M., Calvo E. (2021). Clinical relevance of persistent off-track hill-sachs lesion after Arthroscopic Latarjet procedure. Am J Sports Med.

[12] Calvo E., Delgado C. (2023). Management of off-track Hill-Sachs lesions in anterior glenohumeral instability. J Exp Orthop.

[13] Calvo E., Valencia M., Foruria A.M., Gonzalez J.A. (2022). Recurrence of instability after Latarjet procedure: causes, results and treatment algorithm. EFORT Open Rev.

[14] Connolly J. (1972). Humeral head defects associated with shoulder dislocation: their diagnostic and surgical significance. Instr Course Lect.

[15] Delgado C., Valencia M., Martínez-Catalán N., Calvo E. (2024). Management of the failed Latarjet procedure. J Shoulder Elbow Surg.

[16] Deng Z., Zheng Y., Su J., Chen S., Deng Z., Zhu W. (2023). Open versus arthroscopic latarjet for recurrent anterior shoulder instability: a systematic review and meta-analysis. Orthop J Sports Med.

[17] Di Giacomo G., De Vita A., Costantini A., de Gasperis N., Scarso P. (2014). Management of humeral head deficiencies and glenoid track. Curr Rev Musculoskelet Med.

[18] Di Giacomo G., Itoi E., Burkhart S.S. (2014). Evolving concept of bipolar bone loss and the Hill-Sachs lesion: from "engaging/non-engaging" lesion to "on-track/off-track" lesion. Arthroscopy.

[19] Di Giacomo G., Peebles L.A., Midtgaard K.S., de Gasperis N., Scarso P., Provencher C.M.T. (2020). Risk factors for recurrent anterior glenohumeral instability and clinical failure following primary latarjet procedures: an analysis of 344 patients. J Bone Joint Surg Am.

[20] Ernstbrunner L., Pastor T., Waltenspül M., Gerber C., Wieser K. (2021). Salvage iliac crest bone grafting for a failed latarjet procedure: analysis of failed and successful procedures. Am J Sports Med.

[21] Flurin P.H., Antoni M., Métais P., Aswad R. (2020). Revision of failed Latarjet with the Eden-Hybinette surgical technique. Orthop Traumatol Surg Res.

[22] Gilat R., Lavoie-Gagne O., Haunschild E.D., Golan E.J., Liechti D.J., Yanke A.B. (2020). Outcomes of the Latarjet procedure with minimum 5- and 10-year follow-up: a systematic review. Shoulder Elbow.

[23] Girard M., Dalmas Y., Martinel V., Mansat P., Bonnevialle N. (2022). Arthroscopic latarjet with cortical buttons versus open latarjet with screws: a short-term comparative study. Am J Sports Med.

[24] Gonzalez-Morgado D., Ardebol J., Noble M.B., Galasso L.A., Menendez M.E., Denard P.J. (2025). No difference in external rotation loss after isolated bankart repair, remplissage, or latarjet: a systematic review and meta-analysis. Am J Sports Med.

[25] Han F., Chin B.Y.Y., Tan B.H.M., Lim C.T., Kumar V.P. (2019). Clinical outcomes of the reverse McLaughlin procedure for Hill-Sachs lesions in anterior shoulder instability. J Orthop Surg (Hong Kong).

[26] Itoi E. (2017). 'On-track' and 'off-track' shoulder lesions. EFORT Open Rev.

[27] Katthagen J.C., Anavian J., Tahal D.S., Millett P.J. (2016). Arthroscopic remplissage and open latarjet procedure for the treatment of anterior glenohumeral instability with severe bipolar bone loss. Arthrosc Tech.

[28] Koo S.S., Burkhart S.S., Ochoa E. (2009). Arthroscopic double-pulley remplissage technique for engaging Hill-Sachs lesions in anterior shoulder instability repairs. Arthroscopy.

[29] Mook W.R., Petri M., Greenspoon J.A., Horan M.P., Dornan G.J., Millett P.J. (2016). Clinical and anatomic predictors of outcomes after the latarjet procedure for the treatment of anterior glenohumeral instability with combined glenoid and humeral bone defects. Am J Sports Med.

[30] Patel R.M., Walia P., Gottschalk L., Abdelmalek N., Rizzi A., Provencher M.T. (2016). The effects of latarjet reconstruction on glenohumeral kinematics in the presence of combined bony defects: a cadaveric model. Am J Sports Med.

[31] Plath J.E., Henderson D.J.H., Coquay J., Dück K., Haeni D., Lafosse L. (2018). Does the arthroscopic latarjet procedure effectively correct "Off-Track" hill-sachs lesions?. Am J Sports Med.

[32] Purchase R.J., Wolf E.M., Hobgood E.R., Pollock M.E., Smalley C.C. (2008). Hill-sachs "remplissage": an arthroscopic solution for the engaging hill-sachs lesion. Arthroscopy.

[33] Randelli P., Fossati C., Stoppani C., Evola F.R., De Girolamo L. (2016). Open Latarjet versus arthroscopic Latarjet: clinical results and cost analysis. Knee Surg Sports Traumatol Arthrosc.

[34] Ranne J.O., Sarimo J.J., Heinonen O.J., Orava S.Y. (2013). A combination of Latarjet and Remplissage for treatment of severe glenohumeral instability and bone loss. A case report. J Orthop.

[35] Saliken D., Lavoué V., Trojani C., Gonzalez J.F., Boileau P. (2017). Combined all-arthroscopic hill-sachs remplissage, latarjet, and bankart repair in patients with bipolar glenohumeral bone loss. Arthrosc Tech.

[36] Thangarajah T., Lambert S. (2015). The management of recurrent shoulder instability in patients with epilepsy: a 15-year experience. J Shoulder Elbow Surg.

[37] Valsamis E.M., Kany J., Bonnevialle N., Clavert P., Loriaut P., Sirveaux F. (2020). The arthroscopic Latarjet: a multisurgeon learning curve analysis. J Shoulder Elbow Surg.

[38] Yamamoto N., Itoi E., Abe H., Kikuchi K., Seki N., Minagawa H. (2007). Contact between the glenoid and the humeral head in abduction, external rotation, and horizontal extension: a new concept of glenoid track. J Shoulder Elbow Surg.

[39] Yang J.S., Mazzocca A.D., Cote M.P., Edgar C.M., Arciero R.A. (2016). Recurrent anterior shoulder instability with combined bone loss: treatment and results with the modified latarjet procedure. Am J Sports Med.

